# Hindbrain Adenosine 5-Triphosphate (ATP)-Purinergic Signaling Triggers LH Surge and Ovulation via Activation of AVPV Kisspeptin Neurons in Rats

**DOI:** 10.1523/JNEUROSCI.1496-22.2023

**Published:** 2023-03-22

**Authors:** Naoko Inoue, Safiullah Hazim, Hitomi Tsuchida, Yuri Dohi, Ren Ishigaki, Ai Takahashi, Yuki Otsuka, Koki Yamada, Yoshihisa Uenoyama, Hiroko Tsukamura

**Affiliations:** Laboratory of Animal Reproduction, Graduate School of Bioagricultural Sciences, Nagoya University, Nagoya 464-8601, Japan

**Keywords:** estrogen-positive feedback, GnRH/LH surge, kisspeptin neuron

## Abstract

Ovulation disorders are a serious problem for humans and livestock. In female rodents, kisspeptin neurons in the anteroventral periventricular nucleus (AVPV) are responsible for generating a luteinizing hormone (LH) surge and consequent ovulation. Here, we report that adenosine 5-triphosphate (ATP), a purinergic receptor ligand, is a possible neurotransmitter that stimulates AVPV kisspeptin neurons to induce an LH surge and consequent ovulation in rodents. Administration of an ATP receptor antagonist (PPADS) into the AVPV blocked the LH surge in ovariectomized (OVX) rats treated with a proestrous level of estrogen (OVX + high E2) and significantly reduced the ovulation rate in proestrous ovary-intact rats. AVPV ATP administration induced a surge-like LH increase in OVX + high E2 rats in the morning. Importantly, AVPV ATP administration could not induce the LH increase in *Kiss1* KO rats. Furthermore, ATP significantly increased intracellular Ca^2+^ levels in immortalized kisspeptin neuronal cell line, and coadministration of PPADS blocked the ATP-induced Ca^2+^ increase. Histologic analysis revealed that the proestrous level of estrogen significantly increased the number of P2X2 receptor (an ATP receptor)-immunopositive AVPV kisspeptin neurons visualized by tdTomato in *Kiss1*-tdTomato rats. The proestrous level of estrogen significantly increased varicosity-like vesicular nucleotide transporter (a purinergic marker)-immunopositive fibers projecting to the vicinity of AVPV kisspeptin neurons. Furthermore, we found that some hindbrain vesicular nucleotide transporter-positive neurons projected to the AVPV and expressed estrogen receptor α, and the neurons were activated by the high E2 treatment. These results suggest that hindbrain ATP-purinergic signaling triggers ovulation via activation of AVPV kisspeptin neurons.

**SIGNIFICANCE STATEMENT** Ovulation disorders, which cause infertility and low pregnancy rates, are a serious problem for humans and livestock. The present study provides evidence that adenosine 5-triphosphate, acting as a neurotransmitter in the brain, stimulates kisspeptin neurons in the anteroventral periventricular nucleus, known as the gonadotropin-releasing hormone surge generator, via purinergic receptors to induce the gonadotropin-releasing hormone/luteinizing hormone surge and ovulation in rats. In addition, histologic analyses indicate that adenosine 5-triphosphate is likely to be originated from the purinergic neurons in the A1 and A2 of the hindbrain. These findings may contribute to new therapeutic controls for hypothalamic ovulation disorders in humans and livestock.

## Introduction

Ovulation is an essential process in producing the next generation of mammals. Occurrence and timing of mammalian ovulation are orchestrated by sex hormones, gonadotropins, and their central regulators, such as gonadotropin-releasing hormone (GnRH) and kisspeptin, to ensure the highest possible chance of fertilization. Among these factors, kisspeptin neurons located in the anterior hypothalamus, such as the anteroventral periventricular nucleus (AVPV) and preoptic area (POA), are considered to play a key role in inducing the GnRH/luteinizing hormone (LH) surge and consequent ovulation. *Kiss1* (kisspeptin gene)- and *Gpr54* (kisspeptin receptor gene)*-*deficient mice, rats, and humans are infertile and associated with hypogonadotropic hypogonadism (De Roux et al., 2003; Seminara et al., 2003; Lapatto et al., 2007; Watanabe et al., 2014; Uenoyama et al., 2015, 2021; Tsukamura, 2022). Indeed, *Kiss1* KO rats never show an LH surge even if treated with a preovulatory level of estrogen (Uenoyama et al., 2015). These findings clearly suggest that kisspeptin-GPR54 signaling is essential for induction of the GnRH/LH surge and consequent ovulation. Kisspeptin neurons in the AVPV in rodents and the POA in ruminants and primates are considered to be an estrogen action site in female mammals and are responsible for GnRH/LH surge generation (Adachi et al., 2007; Smith et al., 2009, 2010; Matsuda et al., 2015). Estrogen increases kisspeptin/*Kiss1* expression in AVPV/POA kisspeptin neurons by acting on estrogen receptor α (ERα) expressed in the neurons (Smith et al., 2005; Franceschini et al., 2006; Adachi et al., 2007), and kisspeptin directly stimulates GnRH neurons via GPR54 (Messager et al., 2005). Furthermore, the preovulatory levels of estrogen activate AVPV/POA kisspeptin neurons in rats, goats, and monkeys (Adachi et al., 2007; Watanabe et al., 2014; Matsuda et al., 2015) before the onset of LH surge. However, upstream stimulator(s) that activate AVPV/POA kisspeptin neurons in the presence of preovulatory levels of estrogen have not been fully defined.

To identify novel upstream signal(s) that trigger kisspeptin release and then the GnRH/LH surge, we first analyzed receptor genes exclusively expressed in isolated kisspeptin neurons by RNA-seq analysis of visualized AVPV kisspeptin neurons taken from *Kiss1*-tdTomato heterozygous female rats (Assadullah et al., 2018). The RNA-seq analysis revealed considerable levels of P2X2 receptor (*P2rx2*) mRNA in AVPV kisspeptin neurons of female rats treated with a preovulatory level of estrogen. Importantly, *P2rx2* mRNA was undetectable in arcuate nucleus (ARC) kisspeptin neurons, another population of kisspeptin neurons that is considered to be responsible for tonic (pulsatile) GnRH/LH release in the control of folliculogenesis and steroidogenesis in female mammals (Clarkson et al., 2017; Nagae et al., 2021). The P2X2 receptor is a stimulatory cation channel gated by extracellular adenosine 5-triphosphate (ATP) (Khakh and North, 2012). Thus, the expression of *P2rx2* specifically in AVPV kisspeptin neurons implies that purinergic P2X2 signaling could be specifically involved in the activation of AVPV kisspeptin neurons. Therefore, we hypothesized that ATP-purinergic receptor signaling is a novel upstream stimulator of the AVPV kisspeptin neurons that trigger the GnRH/LH surge and consequent ovulation in female mammals.

Here we demonstrate that ATP-purinergic receptor signaling plays a critical role in the activation of AVPV kisspeptin neurons, considered the GnRH/LH surge generator, to trigger ovulation in rats. Further, we demonstrate that purinergic neurons, which are the source of ATP involved in GnRH/LH surge generation, are likely to originate from A1 and A2 in the hindbrain.

## Materials and Methods

### Animals

Female WT (Wistar-Imamichi strain), *Kiss1* KO, and *Kiss1*-tdTomato heterozygous (*Kiss1*-tdTomato) rats (Uenoyama et al., 2015) (aged 8-16 weeks; 200-320 g body weight) were maintained in a controlled environment (14 h light and 10 h darkness, lights on at 0500 h; 23 ± 3°C) and allowed free access to standard laboratory rat chow (CE-2; Clea) and water. Vaginal smears of WT and *Kiss1*-tdTomato females were checked daily to determine estrous cyclicity, and females having at least two consecutive estrous cycles were used. All animal experiments were approved by the Committee on Animal Experiments of the Graduate School of Bioagricultural Sciences, Nagoya University.

### Ovariectomy and hormone supplementation

Unless otherwise stated, female rats were bilaterally OVX under anesthesia with an intraperitoneal injection of ketamine (26.7 mg/kg) and xylazine (5.3 mg/kg) mixture followed by inhalation of isoflurane (1%-3%). Some OVX rats then immediately received a subcutaneous implant of Silastic tubing (inner diameter 1.57 mm, outer diameter 3.18 mm, and length 25 mm; Dow Corning) filled with E2 (Sigma) dissolved in peanut oil at 20 μg/ml. The implant was in place for 7 d to give OVX + low E2 rats. This E2 treatment was chosen to produce a negative-feedback level of plasma E2 that suppresses LH pulses (diestrous model) (Cagampang et al., 1990). OVX + high E2 rats (proestrous model) were produced by a subcutaneous implant of Silastic tubing (28 mm in length) filled with E2 dissolved in peanut oil at 1000 μg/ml (high E2) for 2 d in OVX rats, which had been implanted with the low E2 tubing for 5 d. This sequential E2 treatment was chosen to produce a positive-feedback level of plasma E2 to induce the LH surge (Nagae et al., 2021).

### Cannula implantation into the AVPV for administration of P2X receptor antagonist or ATP

Female rats anaesthetized in the same manner were stereotaxically implanted with a stainless-steel guide cannula (C315G, Plastics One) into the AVPV with its tip at 0.12 mm posterior to bregma, 0.5 mm from the midline, and 8.0 mm below the surface of the skull according to the coordinates of a rat brain atlas (Paxinos and Watson, 2008). On the same day, the animals were ovariectomized and received E2 implants, as described above.

### Effect of P2X receptor antagonist administration into AVPV on LH surge and ovulation

Seven days after brain surgery, blood samples (100 μl) were collected every 1 h (10:00-21:00 h) through a silicon cannula (inner diameter 0.5 mm and outer diameter 1.0 mm; Shin-Etsu Polymer) inserted into the right atrium via the jugular vein on the day before the blood sampling to determine whether AVPV injection of a P2X receptor antagonist (pyridoxalphosphate-6-azophenyl-2′,4′-disulfonic acid [PPADS], 15 nmol/1.5 μl, Tocris Bioscience) blocks the E2-induced LH surge in WT OVX + high E2 rats (*n* = 4). PPADS or vehicle (aCSF: 128 mm NaCl, 3.0 mm KCl, 1.2 mm CaC1_2_, 0.8 mm MgC1_2_, 0.65 mm NaH_2_PO_4_, and 4.8 mm NaHCO_3_, pH 7.0; *n* = 6) was injected into the AVPV through an inner cannula (C315I, Plastics One) inserted into the guide cannula using a microsyringe pump (Eicom) with a flow rate of 1.5 µl/15 min immediately after the third blood sampling at 12:00 h. Plasma samples (50 μl) were obtained by immediate centrifugation and stored at −20°C until assayed for LH.

We also determined whether PPADS administration into the AVPV attenuates ovulation in ovary-intact WT female rats. After the brain surgery, estrous cyclicity was daily monitored for at least 6 d and blood was collected every 1 h (10:00-21:00 h) at proestrus. One day after the PPADS (*n* = 6) or vehicle (*n* = 4) administration and blood sampling, the number of ovulated oocytes in the oviducts was counted under a stereomicroscope.

### Effect of AVPV ATP administration on plasma LH levels in WT and *Kiss1* KO female rats

Seven days after the brain surgery, blood samples were collected every 6 min for 3 h (10:00-13:00 h) to determine the effect of AVPV injection of the P2X2 receptor agonist, ATP (200 nmol/0.5 μl; Sigma-Aldrich), on LH secretion in OVX + high E2 WT (*n* = 6) and *Kiss1* KO (*n* = 5) rats (Uenoyama et al., 2015). ATP or vehicle (ultra-pure water; WT rats, *n* = 4 and *Kiss1* KO rats, *n* = 5) was injected as described above with a flow rate of 0.5 µl/4 min immediately after the first blood sampling at 10:00 h. Red blood cells taken from donor rats and washed with heparinized saline were replaced at each blood collection to keep the hematocrit level constant.

### Radioimmunoassay for LH

Plasma LH concentrations were measured by a double-antibody radioimmunoassay, as previously described (Watanabe et al., 2017), using a rat LH RIA kit provided by the National Hormone and Peptide Program (Harbor-UCLA Medical Center), and are expressed in terms of National Institute of Diabetes and Digestive and Kidney Diseases rat LH-reference preparation (RP)−3. The lowest detectable LH concentration was 39 pg/tube, and the intra- and inter-assay and interassay coefficients of variation were 5.6% at 2.3 ng/ml and 6.4% at 1.4 ng/ml, respectively.

### Brain sampling for immunohistochemistry

Brain samples were collected at 13:00-15:00 h for single immunohistochemical staining of P2X2 receptor or VNUT, or double immunohistochemical staining of VNUT and GnRH, VNUT and ERα, or VNUT and dopamine β-hydroxylase (DBH) in OVX + high E2 WT rats. The timing of brain sampling was chosen because the E2-induced LH surge usually starts at this time in OVX + high E2 rats. To investigate activated purinergic neurons, brain samples were collected at 11:00-12:00 h for double immunohistochemical staining of VNUT and c-Fos. This timing was chosen because it was just before the onset of E2-induced LH surge in OVX + high E2 rats. Briefly, animals were deeply anesthetized with sodium pentobarbital (40 mg/kg) and perfused with 4% PFA. Brains were postfixed with the same fixative. According to a rat brain atlas (Paxinos and Watson, 2008), serial 50 μm coronal sections of the hypothalamus, including the AVPV (from 0.48 mm anterior to 0.24 mm posterior to bregma), POA/medial septum-diagonal band of Broca (MSDB) (from 0.60 mm anterior to 0.84 mm posterior to bregma), suprachiasmatic nucleus (SCN) (from 0.48 mm to 0.96 mm posterior to bregma), periventricular hypothalamic nucleus (Pe) (from 0.48 mm to 1.56 mm posterior to bregma), ARC (from 1.72 mm to 4.36 mm posterior to bregma), paraventricular nucleus dorsal cap (PaDC) (from 1.72 mm posterior to 1.92 mm posterior to bregma), and supramammillary nucleus medial part (SuM) (from 4.20 mm posterior to 4.92 mm posterior to bregma), and of the hindbrain, including A6 (from 9.48 mm posterior to 10.20 mm posterior to bregma), area postrema (AP) (from 13.68 mm posterior to 14.28 mm posterior to bregma), A1 (from 14.72 mm posterior to 15.96 mm posterior to bregma), and A2 (from 14.64 mm posterior to 15.96 mm posterior to bregma) were cut on a cryostat (CM1800, Leica Biosystems) and subjected to immunohistochemical analyses.

### P2X2 receptor immunohistochemistry

To examine whether P2X2 receptors are located in AVPV kisspeptin neurons, P2X2 receptor immunohistochemistry was performed on hypothalamic sections containing the AVPV region of OVX + low E2 and OVX + high E2 *Kiss1*-tdTomato rats (*n* = 4 in each group), in which kisspeptin neurons are visualized by tdTomato fluorescence as previously reported (Uenoyama et al., 2015). Every second section was incubated with an anti-P2X2 receptor antibody (1:2000; Alomone Labs; RRID:AB_2040054) over 7 nights at 4°C. The sections were then incubated with a peroxidase-conjugated anti-rabbit IgG antibody (1:1000; Vector Laboratories; RRID:AB_2336198) over 3 nights at 4°C, and then processed using a Tyramide Signal Amplification Plus Biotin kit (1:100, PerkinElmer) for 2 h at room temperature (RT), according to the manufacturer's instructions. The sections were then incubated with AlexaFluor-488-conjugated streptavidin (1:1000; Fisher Scientific) for 2 h at RT.

### VNUT immunohistochemistry

To investigate the projection of purinergic neurons to kisspeptin neurons, VNUT immunohistochemistry was performed on hypothalamic sections, including the AVPV region taken from OVX + low E2 or OVX + high E2 *Kiss1*-tdTomato female rats (*n* = 5 in each group). Every second section was incubated with an anti-VNUT antibody (1:2000; Millipore; RRID:AB_2868445) over 7 nights at 4°C and then incubated with an Alexa-488-conjugated anti-guinea pig IgG antibody (1:800; Jackson ImmunoResearch Laboratories; RRID:AB_2340472) for 2 h at RT.

### GnRH and VNUT double immunohistochemistry

To investigate the projection of purinergic neurons to GnRH neurons, double immunohistochemistry for VNUT and GnRH was performed on hypothalamic sections that included the POA/MSDB taken from OVX + high E2 WT rats (*n* = 3). Every second section was incubated with the anti-VNUT antibody (1:2000) over 7 nights at 4°C and then incubated with the anti-GnRH antibody [1:2000; kindly donated by Dr. Park (Park and Wakabayashi, 1986); RRID:AB_2636958] over 1 night at RT. The sections were then incubated with the Alexa-488-conjugated anti-guinea pig IgG antibody (1:800) for VNUT and an Alexa-594-conjugated anti-mouse IgG antibody (1:800; Fisher Scientific; RRID:AB_141633) for GnRH for 2 h at RT.

### VNUT and C-Fos double immunohistochemistry

To investigate the activation of purinergic neurons, VNUT and c-Fos double immunohistochemistry was performed on hypothalamic sections that included the Pe, PaDC, ARC, and SuM, and on hindbrain sections that included the A1, A2, A6, and AP, taken from OVX + low E2 and OVX + high E2 WT rats (*n* = 3 in each group). Every fourth section was incubated with the anti-VNUT antibody (1:2000) and an anti-c-Fos antibody (1:4000; Millipore; RRID:AB_2106755) over 7 nights at 4°C. The sections were then incubated with the Alexa-488-conjugated anti-guinea pig IgG antibody (1:800) for VNUT and an Alexa-594-conjugated anti-rabbit IgG antibody (1:800; Fisher Scientific; RRID:AB_141637) for c-Fos for 2 h at RT.

### VNUT and Fluorogold (FG) double immunohistochemistry

To investigate the projection of purinergic neurons to the AVPV, FG (Biotium), a retrograde tracer, was unilaterally injected into the AVPV of OVX + high E2 WT rats (*n* = 4) at a rate of 0.5 µl/min for 10 s. Brain samples were taken 2 weeks after the tracer injection, and brain sections of the hypothalamus and hindbrain were made as described above. To visualize FG and VNUT signals, the brain sections were incubated with a guinea pig anti-VNUT antibody (1:2000) and an anti-FG antibody (1:3000; Millipore; RRID:AB_90738) for 48 h at 4°C, and the sections were then incubated with the AlexaFluor-488-conjugated anti-guinea pig IgG antibody (1:800) and the Alexa-594-conjugated anti-rabbit IgG antibody (1:800) for 2 h at RT.

### VNUT and ER alpha double immunohistochemistry

To investigate whether ERα is expressed in purinergic neurons, VNUT and ERα double immunohistochemistry was performed on hypothalamic and hindbrain sections taken from OVX WT rats (*n* = 3). OVX rats were used because the presence of high levels of E2 reduces ERα immunoreactivity with this anti-ERα antibody in our preliminary experiment. The sections were incubated with the anti-VNUT antibody (1:2000) and an anti-ERα antibody (1:1000; Millipore; RRID:AB_310105) over 7 nights at 4°C. The sections were then treated with the Alexa-488-conjugated anti-guinea pig IgG antibody (1:800) for VNUT and Alexa-594-conjugated anti-rabbit IgG antibody (1:800) for ERα for 2 h at RT.

### VNUT and DBH double immunohistochemistry

To confirm whether the brainstem purinergic neurons are noradrenergic neurons, VNUT and DBH double immunohistochemistry was performed on hindbrain sections taken from OVX + low E2 and OVX + high E2 WT rats (*n* = 3 in each group). The sections were incubated with the anti-VNUT antibody and an anti-DBH antibody (1:2000; Millipore; RRID:AB_2245740) over 7 nights at 4°C. The sections were then incubated with the Alexa-488-conjugated anti-guinea pig IgG antibody (1:800) for VNUT and the Alexa-594-conjugated anti-mouse IgG antibody (1:800) for DBH for 2 h at RT.

### Quantification of immunoreactive cells and fibers

Images of immunofluorescent cells and tdTomato red fluorescence were taken using a fluorescence microscope (Zeiss) with ApoTome. Positive cell bodies were counted at least twice in every second section through the AVPV, POA/MSDB (unilaterally), and in every fourth section through the Pe, ARC, PaDC, A1, A2, A6 (unilaterally), and the SuM and AP (bilaterally), and the average was calculated. For analysis of purinergic neuron projections to tdTomato-positive kisspeptin or GnRH neurons, the total number of tdTomato-positive or GnRH-immunoreactive cell bodies, the number of tdTomato-positive or GnRH-immunoreactive cell bodies with contact with VNUT-immunoreactive fibers, and the number of VNUT-immunoreactive fibers on tdTomato-positive or GnRH-immunoreactive cell bodies were unilaterally counted twice and the average was calculated. For analysis of retrograde tracing using AVPV FG injection, the number of FG and/or VNUT-positive cells was counted twice in the hypothalamus and hindbrain regions ipsilateral to the FG injection side, and the average was calculated.

### Culture of a mouse AVPV kisspeptin neuron-derived immortalized cell line and calcium imaging

An immortalized mouse neuronal cell line, mHypoA-51 (#CLU460; CELLutions Biosystems), was used as a model of AVPV kisspeptin neurons (Belsham et al., 2004; Mittelman-Smith et al., 2015) to investigate direct effects of ATP on activation of AVPV kisspeptin neurons. The cells were cultured in DMEM (Sigma-Aldrich) supplemented with 1% penicillin/streptomycin and 10% FBS (Biowest). For calcium imaging and analysis of *P2rx2* mRNA by real-time RT-PCR, the cells were cultured with phenol red-free DMEM (Fisher Scientific) supplemented with 4 mm L-glutamine, 1% penicillin/streptomycin, 5% charcoal-stripped FBS, and E2 (0 or 1 nm) for 4 h. This E2 treatment was based on a report by Mittelman-Smith et al. (2015) showing that treatment with 1 nm E2 for 24 h increased *Kiss1* expression in mHypoA-51 cells, and our preliminary experiments using the same cell line showed an increase in *Kiss1* expression on 1 nm E2 treatment for 4 h, but not 24 h.

Intracellular Ca^2+^ imaging was performed with fura-2 AM (AAT Bioquest) using a microscope (IX81, Olympus) equipped with a progressive scan interline CCD camera C13440-20CU (Hamamatsu Photonics). Three to four days before imaging, mHypoA-51 cells (0.72-1.44 × 10^3^ cells/ml) were plated onto poly-D-lysine (0.1 mg/ml; Sigma-Aldrich)-coated 35 mm glass-base dishes (Iwaki) and grown in DMEM with 10% FBS and 1% penicillin/streptomycin at 37°C in 5% CO_2_. A day before imaging, the medium was changed to DMEM supplemented with 5% charcoal-stripped FBS (Biowest), 1% penicillin/streptomycin, glutamine, and sodium bicarbonate. Four and a half hours before imaging, the medium was changed to phenol red-free DMEM (Fisher Scientific) supplemented with 4 mm L-glutamine, 1% penicillin/streptomycin, 5% charcoal-stripped FBS, and 1 nm E2. Before imaging, cells were loaded with the calcium indicator, fura-2 AM (5 μm), dissolved in Krebs-Ringer bicarbonate buffer containing 0.02% Pluronics F-127 (Biotium), and 0.05% DMSO for 25 min at 37°C. They were then superfused with Krebs-Ringer bicarbonate buffer containing ATP (10 μm, Sigma-Aldrich) or PPADS (100 μm, Tocris Bioscience). Viability of cells was determined by a Ca^2+^ increase in response to 100 mm KCl at the end of Ca^2+^ measurement. Fluorescence images were taken every 5 s with excitation wavelengths of 340 and 380 nm. Ratio images were obtained by MetaMorph Meta Imaging Series (version 7.10, Molecular Devices), processed by ImageJ software (version 1.5.0, http://imagej.nih.gov/ij/), and analyzed by MATLAB software (The MathWorks). Briefly, ratio data (fluorescent levels excited by the wavelength at 340 nm to those at 380 nm) were standardized by cubic baseline subtraction, and the area under the curve (AUC) of intracellular Ca^2+^ levels was calculated using the Gaussian function as reported previously (Minabe et al., 2015).

### Real-time RT-PCR analysis

DNA-free total RNA was purified from mHypoA-51 cells using ISOGEN (Nippon Gene). ReverTra Ace (Toyobo) was used to synthesize full-length cDNAs, which were used as templates in PCRs using primers for *P2rx2* and *Actb*. Forward and reverse primers for mouse *P2rx2*, 5′-GCGTTCTGGGACTACGAGAC-3′ and 5′-GATCCCCTTGACTTTGGTGA-3′ (GenBank accession no. AY044240.2) and for mouse *Actb*, 5′-GGTGGGAATGGGTCAGAAGG-3′ and 5′-GTACATGGCTGGGGTGTTGA-3′ (GenBank accession no. NM_007393.5). Real-time PCR analysis was performed using an ABI 7500 real-time system (Fisher Scientific) with Thunderbird qPCR Mix (Toyobo). The cycling protocol was as follows: pre-denaturation for 1 min at 95°C, 40 amplification cycles of 15 s at 95°C, and 1 min at 60°C. The specificity of the amplification products was confirmed by dissociation curve analysis (60°C-95°C) after the 40 cycle amplification. A distinct single peak was considered to confirm that only a single DNA sequence was amplified. The expression levels of *P2rx2* were obtained by normalization to the expression levels of *Actb*.

### Analysis of RNA-seq data from *Kiss1*-tdTomato heterozygous rats

Expression profiles of genes encoding purinergic receptors in AVPV or ARC kisspeptin neurons were analyzed using RNA-seq data from isolated AVPV or ARC kisspeptin neurons from *Kiss1*-tdTomato heterozygous female rats as described previously (Assadullah et al., 2018).

### Experimental design and statistical analysis

The sample size for each experiment is given in Results. Values given in the text and illustrated in figures are mean ± SEM. Statistical differences in the AUC of plasma LH concentrations after the administration of P2X receptor antagonist (PPADS), and in the number of ovulated oocytes were determined by Student's *t* test. Statistical differences in the AUC for plasma LH concentrations after ATP administration between groups were determined by two-way [ATP injection and *Kiss1* expression (WT or *Kiss1* KO rats) as main effects] ANOVA. Statistical differences in the AUC of intracellular Ca^2+^ levels between groups were determined by two-way [E2 treatment and time (see Fig. 3*B*) or PPADS injection and time (see Fig. 3*E*) as main effects] repeated-measures ANOVA. Statistical differences in *P2rx2* expression in mHypoA-51 cells, and in the number of the cells or fibers examined by histologic analyses between groups were determined by Student's *t* test. All analyses were performed using SAS University Edition (https://www.sas.com/ja_jp/software/university-edition.html).

## Results

### *P2rx2* expression in AVPV kisspeptin neurons determined by RNA-seq analysis

To identify novel signals upstream of AVPV kisspeptin neurons, we first investigated receptor genes specifically expressed in AVPV kisspeptin neurons. RNA-seq analysis of AVPV kisspeptin neurons isolated from *Kiss1*-tdTomato heterozygous female rats revealed expression of approximately 6300 genes according to Reads Per Kilobase of transcript per Million mapped reads (RPKM) values (total reads: 100,354,874, mean read length: 53 bp, aligned base rate: 90%). The RNA-seq analysis of ARC kisspeptin neurons showed expression of approximately 8200 genes (total reads: 66,840,006, mean read length: 53 bp, aligned base rate: 88%). As shown in Table 1, *P2rx2* was highly expressed in the AVPV kisspeptin neurons of OVX + highE2 *Kiss1*-tdTomato rats, but not in ARC kisspeptin neurons in OVX *Kiss1*-tdTomato rats. Importantly, gene expression of other purinergic receptors, such as P1, P2X, and P2Y receptors, were undetectable in the AVPV kisspeptin neurons of *Kiss1*-tdTomato rats. Thus, we focused on the role of P2RX2 in AVPV kisspeptin neurons.

**Table 1.** Gene-expression profiles of purinergic receptors analyzed by RNA seq in the visualized AVPV and ARC *Kiss1* cells taken from *Kiss1*-tdTomate female rats

Purinergic receptors	Genes	OVX + high E2 AVPV (RPKM)	OVX ARC (RPKM)
P1 receptors	Adora1	0	0
	Adora2a	0	0
	Adora2b	0	0
	Adora3	0	0
P2X receptors	P2rx1	0	0
	P2rx2	272.41	0
	P2rx3	0	0
	P2rx4	0	0
	P2rx5	0	0
	P2rx6	0	0
	P2rx7	0	0
P2Y receptors	P2ry1	0	0
	P2ry2	0	0.11
	P2ry4	0	0
	P2ry6	0	0
	P2ry10	0	0
	P2ry12	0	0
	P2ry13	0	0

### AVPV P2X receptor antagonism blocked estrogen-induced LH surge and AVPV ATP administration induces a surge-like LH increase in female rats

**Figure 1.**
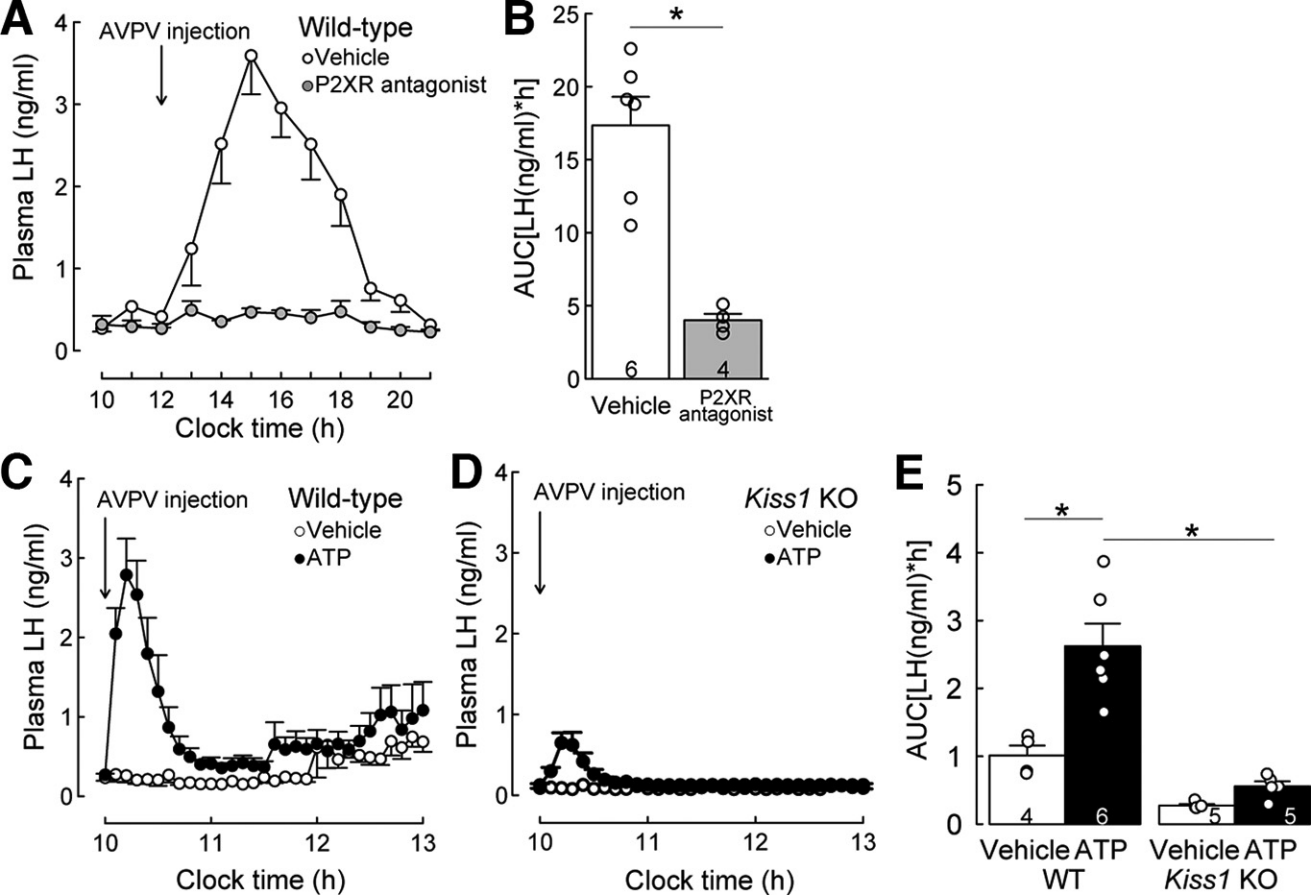
ATP-P2X receptor signaling in the AVPV mediates estrogen-induced LH surge via activation of kisspeptin neurons in female rats. ***A***, The AVPV administration of PPADS, a P2X receptor (P2XR) antagonist, completely blocked the E2-induced endogenous LH surge in OVX WT rats, while the E2-induced LH surge was apparent in the afternoon in the vehicle-treated control OVX + high E2 WT rats. ***B***, The AUC of plasma LH levels was significantly lower in AVPV P2XR antagonist (PPADS)-treated WT rats (*n* = 4) compared with that of vehicle-treated controls (*n* = 6). *p = 0.0007 (Student's *t* test). ***C***, ATP administration into the AVPV immediately induced a surge-like increase in plasma LH levels in OVX + high E2 WT rats. ***D***, AVPV ATP administration failed to induce a surge-like increase in plasma LH levels in OVX + high E2 *Kiss1* KO rats. ***E***, The AUC of plasma LH levels was significantly higher in AVPV ATP-treated rats (*n* = 6) compared with that in vehicle-treated control rats in WT (*n* = 4, *p < 0.0001, the simple main effect of the two-way ANOVA), but not *Kiss1* KO rats (*n* = 5, *p* > 0.05, the simple main effect of the two-way ANOVA). The AUC of plasma LH levels after AVPV ATP administration in OVX + high E2 WT rats (*n* = 6) was significantly higher compared with that in OVX + high E2 *Kiss1* KO rats (*n* = 5). *p < 0.0001 (the simple main effect of the two-way ANOVA). Two-way ANOVA revealed significant main effects of ATP injection [*F*_(1,16)_ = 41.48, *p* < 0.0001] and *Kiss1* expression [*F*_(1,16)_ = 18.88, *p* = 0.0005], as well as a significant interaction between the main effects [*F*_(1,16)_ = 9.27, *p* = 0.0077]. Arrow indicates the onset of administration. Data are mean ± SEM.

We investigated the effect of P2X receptor antagonism in the AVPV region on the endogenous LH surge to determine whether AVPV P2X receptors mediate estrogen-positive feedback to induce the LH surge. The AVPV administration of PPADS, a P2X receptor antagonist, completely blocked the estrogen-induced endogenous LH surge in OVX + high E2 WT rats (Fig. 1*A*), while the estrogen-induced LH surge was apparent in the afternoon in vehicle-treated control OVX + high E2 WT rats (Fig. 1*A*). The AUC of plasma LH levels was significantly lower in the AVPV PPADS-treated rats (*n* = 4) compared with that of vehicle-treated controls (*n* = 6, *p* = 0.0007, Fig. 1*B*).

An administration of ATP, a major ligand of the P2X2 receptor, into the AVPV immediately induced a surge-like increase in plasma LH levels in OVX + high E2 WT rats in the morning, when the endogenous LH surge never occurs (Fig. 1*C*), and the AUC of plasma LH levels was significantly higher in AVPV ATP-treated rats (*n* = 6) compared with that in vehicle-treated control OVX + high E2 WT rats (*n* = 4, *p* < 0.0001, Fig. 1*E*). To clarify whether the AVPV ATP-induced LH increase was mediated by kisspeptin neurons, changes in LH release after the AVPV ATP challenge were investigated in global *Kiss1* KO female rats. The AVPV ATP administration failed to induce a surge-like increase in LH levels in OVX + high E2 *Kiss1* KO rats (Fig. 1*D*), resulting in no significant difference between AVPV ATP-treated and vehicle-treated *Kiss1* KO rats (*n* = 5, Fig. 1*E*). The AUC of plasma LH levels after AVPV ATP administration to OVX + high E2 WT rats was significantly higher compared with that of OVX + high E2 *Kiss1* KO rats (*p* < 0.0001, Fig. 1*E*).

### AVPV P2X receptor antagonism attenuated spontaneous LH surge and ovulation in proestrous rats

**Figure 2.**
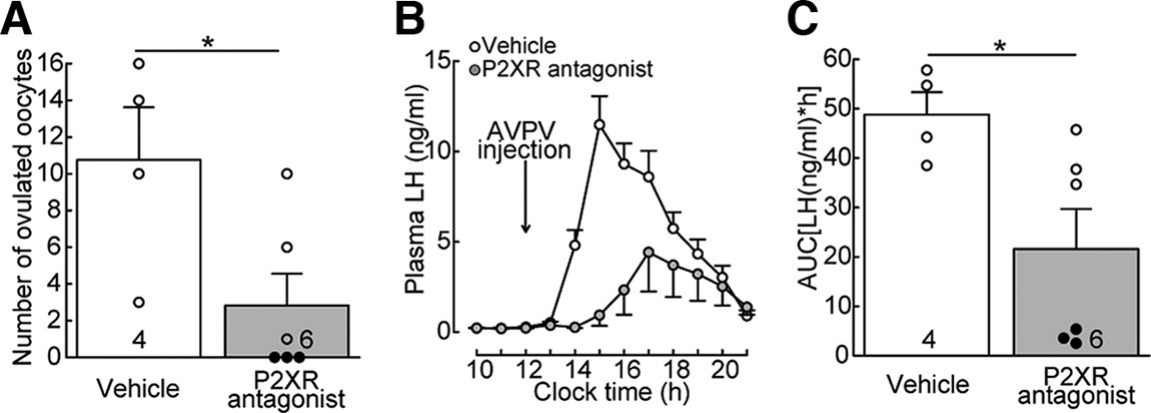
AVPV P2X receptor antagonism suppresses spontaneous LH surge and ovulation in female rats. ***A***, AVPV administration of P2XR antagonist (PPADS) profoundly reduced the spontaneous LH surge in proestrous rats. ***B***, The AUC of plasma LH levels was significantly lower in AVPV P2XR antagonist (PPADS)-treated proestrous rats (*n* = 6) compared with that of vehicle-treated controls (*n* = 4). *p = 0.0349 (Student's *t* test). ***C***, AVPV administration of P2XR antagonist (PPADS) blocked ovulation in 3 (indicated by solid circles) of 6 proestrous rats, whereas all proestrous rats (*n* = 4) showed ovulation when vehicle was administered into the AVPV. The number of ovulated ova was significantly lower in AVPV P2XR antagonist (PPADS)-administered proestrous rats (*n* = 6) compared with vehicle-administered control rats (*n* = 4). *p = 0.0351 (Student's *t* test). Arrow indicates the onset of administration. Data are mean ± SEM.

Next, we investigated whether PPADS administration blocks spontaneous ovulation. The AVPV administration of PPADS profoundly reduced the endogenous LH surge in proestrous rats (Fig. 2*A*), such that the LH surge was only partially observed in the afternoon (Fig. 2*A*). As a result, the AUC of plasma LH levels was significantly lower in the AVPV PPADS-treated proestrous rats (*n* = 6) compared with that of vehicle-treated controls (*n* = 4, *p* = 0.0349, Fig. 2*B*). Administration of PPADS into the AVPV region blocked ovulation in 3 of 6 proestrous WT ovary-intact female rats (Fig. 2*C*), whereas all vehicle-treated proestrous rats (*n* = 4) showed ovulation. The number of ovulated ova was significantly lower in the AVPV PPADS-administered proestrous rats compared with vehicle-administered control rats (*p* = 0.0351, Fig. 2*A*).

### *In vitro* administration of ATP increased intracellular Ca^2+^ levels via P2X receptor in a mouse AVPV kisspeptin neuronal cell line (mHypoA-51)

**Figure 3.**
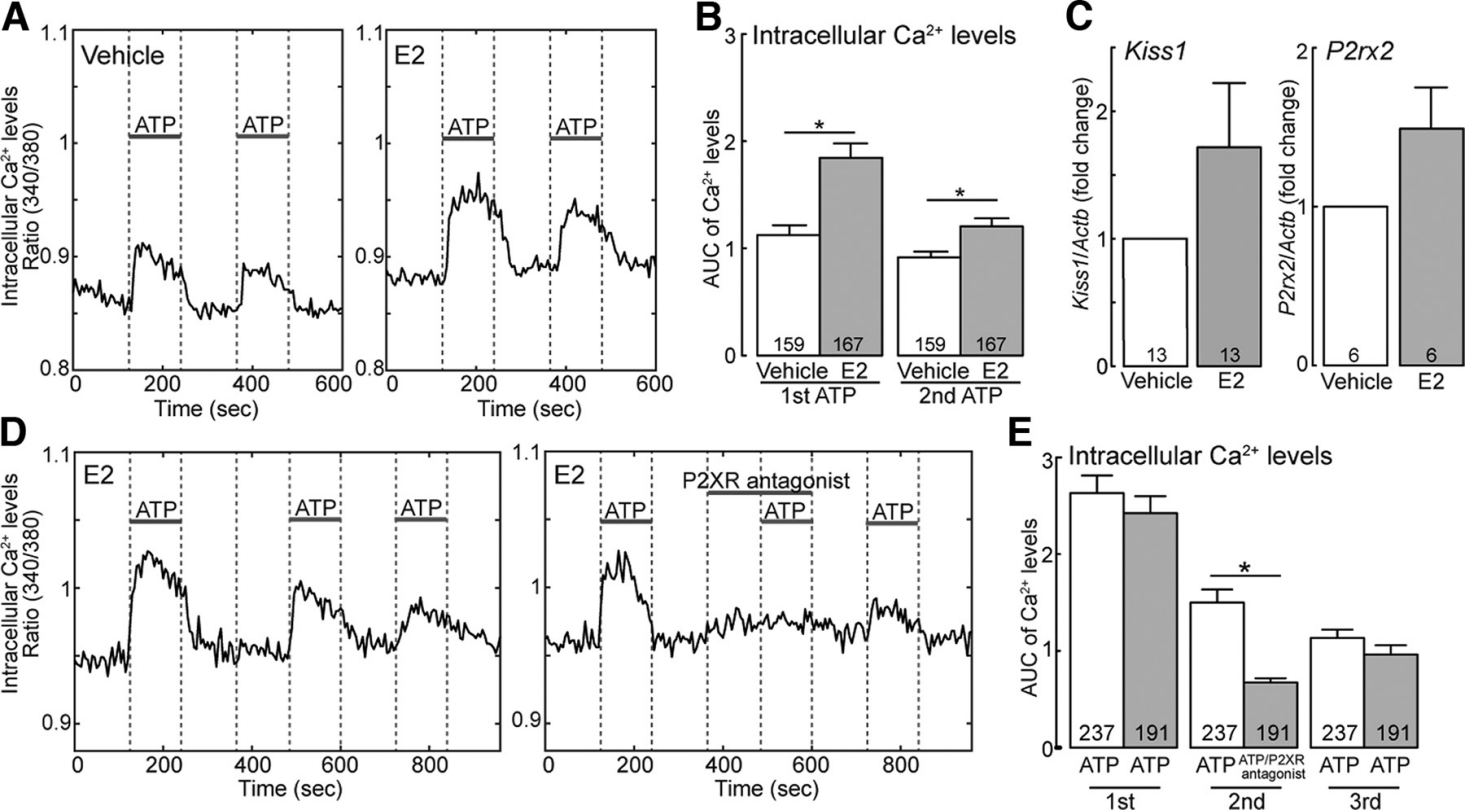
ATP-P2X receptor signaling activates mHypoA-51 cells as a model of rodent AVPV kisspeptin neurons. ***A***, Each *in vitro* ATP administration immediately increased intracellular Ca^2+^ levels in vehicle-pretreated or estrogen-pretreated mHypoA-51 cells. ***B***, The AUC of the Ca^2+^ levels induced by the first and second ATP administration were significantly higher in estrogen-pretreated mHypoA-51 cells (*n* = 167) compared with that of vehicle-pretreated cells (*n* = 159). *p < 0.0001, first ATP; *p = 0.0019, second ATP; the simple main effect of the two-way ANOVA. Two-way ANOVA revealed significant main effects of E2 treatment [*F*_(1,324)_ = 17.23, *p* < 0.0001] and time [*F*_(1,324)_ = 65.18, *p* < 0.0001], as well as a significant interaction between the main effects [*F*_(1,324)_ = 16.49, *p* < 0.0001]. ***C***, *Kiss1* and *P2rx2* mRNA levels tended to be increased by estrogen pretreatment of mHypoA-51 cells (*Kiss1*, *n* = 13, *p* = 0.1854; *P2rx2*, *n* = 6, *p* = 0.2488, Student's *t* test). ***D***, Each ATP administration (3 in total) induced an intracellular Ca^2+^ increase in estrogen-pretreated mHypoA-51 cells, whereas P2XR antagonist (PPADS) treatment, prior to the second administration of ATP, blocked the ATP-induced increase in Ca^2+^ levels. ***E***, P2XR antagonist (PPADS) treatment of mHypoA-51 cells significantly decreased the AUC of intracellular Ca^2+^ levels at the second administration of ATP (*n* = 191) compared with no P2XR antagonist (PPADS) treatment (*n* = 237). *p < 0.0001 (the simple main effect of the two-way ANOVA). Two-way ANOVA revealed significant main effects of P2XR antagonist (PPADS) treatment [*F*_(1,426)_ = 6.08, *p* = 0.0141] and time [*F*_(2,852)_ = 218.24, *p* < 0.0001], as well as a significant interaction between the main effects [*F*_(2,852)_ = 10.50, *p* < 0.0001]. Data are mean ± SEM.

We examined whether ATP-P2X signaling activates AVPV kisspeptin neurons *in vitro* using an immortalized mouse neuronal cell line (mHypoA-51) as a model of AVPV kisspeptin neuron to investigate whether ATP can activate AVPV kisspeptin neurons. As shown in representative profiles of intracellular Ca^2+^ levels in mHypoA-51 cells (Fig. 3*A*), each ATP administration immediately increased intracellular Ca^2+^ levels in vehicle-pretreated and estrogen-pretreated mHypoA-51 cells. Notably, the AUC of the Ca^2+^ levels induced by the first and second ATP administration was significantly higher in estrogen-pretreated mHypoA-51 cells (*n* = 167) compared with that of vehicle-pretreated cells (*n* = 159; *p* < 0.0001, first ATP administration; *p* = 0.0019, second ATP administration; Fig. 3*B*). *Kiss1* and *P2rx2* mRNA levels tended to be increased by estrogen pretreatment (*Kiss1*, *n* = 13, *p* = 0.1854; *P2rx2*, *n* = 6, *p* = 0.2488, Fig. 3*C*). Furthermore, each ATP administration (3 in total) induced an intracellular Ca^2+^ increase in estrogen-pretreated mHypoA-51 cells (Fig. 3*D*), whereas treatment with PPADS, a P2X receptor antagonist, before the second administration of ATP blocked the ATP-induced increase in Ca^2+^ levels in mHypoA-51 cells. The third administration of ATP without PPADS induced an immediate increase in intracellular Ca^2+^ in the mHypoA-51 cells (Fig. 3*D*). Statistical analysis revealed that PPADS treatment of mHypoA-51 cells significantly decreased the AUC of intracellular Ca^2+^ levels at the second administration of ATP (*n* = 191) compared with non–PPADS-treated cells (*n* = 237, *p* < 0.0001, Fig. 3*E*). There was no significant difference in the AUC of intracellular Ca^2+^ levels after the first or third administration of ATP without PPADS treatment between groups (Fig. 3*E*).

### AVPV kisspeptin neurons express P2X2 receptors

**Figure 4.**
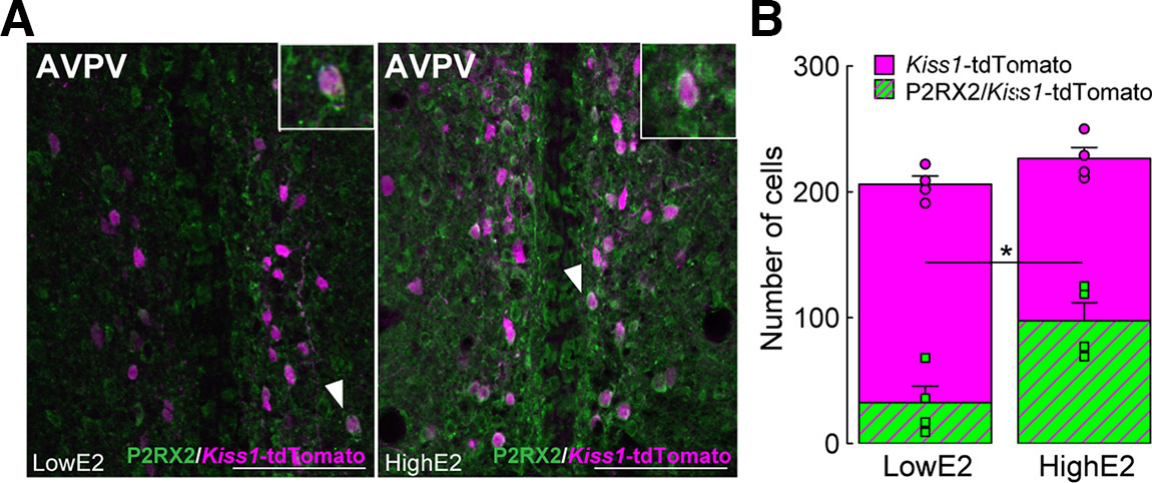
Proestrous levels of E2 induce P2X2 receptor expression in AVPV kisspeptin neurons in female rats. ***A***, P2RX2-immunopositive signals were found on the surface of tdTomato-positive kisspeptin neurons in the AVPV of OVX kisspeptin-visualized-tdTomato female rats treated with low E2 (diestrous model) or high E2 (proestrous model). ***B***, The number of P2RX2-coexpressing tdTomato-positive cells in OVX + high E2 *Kiss1*-tdTomato rats (*n* = 4) was significantly higher than that in OVX + low E2 *Kiss1*-tdTomato rats (*n* = 4). *p = 0.0159 (Student's *t* test). Data are mean ± SEM. Scale bars, 100 μm.

P2RX2-immunopositive signals were found on the surface of tdTomato-positive kisspeptin neurons in the AVPV of OVX *Kiss1-*tdTomato female rats treated with low E2 (diestrous model) or high E2 (proestrous model) (Fig. 4*A*). The number of P2RX2-expressing tdTomato-positive cells in the OVX + high E2 *Kiss1*-tdTomato rats (*n* = 4) was significantly higher than that in OVX + low E2 *Kiss1*-tdTomato rats (*n* = 4, *p* = 0.0159, Fig. 4*B*).

### Purinergic neuronal projection to AVPV kisspeptin neurons

**Figure 5.**
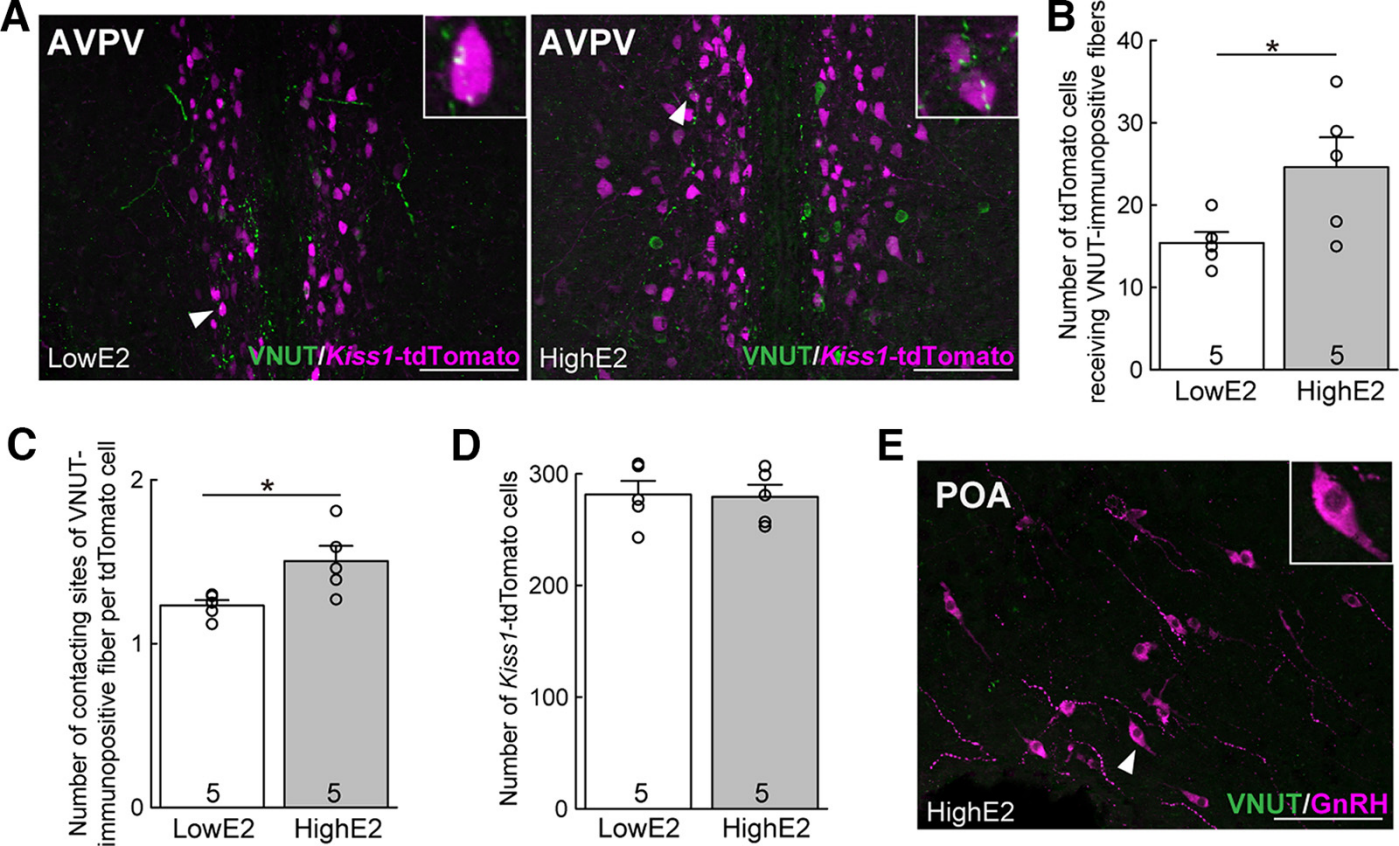
Purinergic neuronal projection to AVPV kisspeptin neurons in female rats. ***A***, VNUT-immunopositive fibers contacted kisspeptin neurons in representative OVX + high E2 and OVX + low E2 *Kiss1*-tdTomato female rats. The number of tdTomato-positive kisspeptin neurons contacted by VNUT-immunopositive fibers (***B***) and the number of VNUT-immunopositive fiber contact sites per tdTomato-positive cell (***C***) were significantly higher in OVX + high E2 *Kiss1*-tdTomato rats compared with those in OVX + low E2 *Kiss1*-tdTomato rats (*n* = 5 in each group): *p = 0.045 (***B***); *p = 0.0256 (***C***); Student's *t* test. ***D***, There was no significant difference in the number of tdTomato-positive kisspeptin neurons between groups. ***E***, No contact of VNUT-immunopositive fibers on GnRH-immunopositive cells was found in the POA in female rats. Data are mean ± SEM. Scale bars, 100 μm.

Next, we examined the projection of purinergic neurons in female rats by immunohistochemistry for VNUT (a marker of purinergic neurons) to confirm that purinergic VNUT-immunoreactive fibers project to AVPV kisspeptin neurons or GnRH neurons in the POA/MSDB. VNUT-immunopositive fibers with varicosity-like structures were found in the AVPV of OVX + high E2 and OVX + low E2 *Kiss1*-tdTomato female rats (Fig. 5*A*). Some VNUT-positive fibers contacted kisspeptin neurons in OVX + high E2 and OVX + low E2 *Kiss1*-tdTomato female rats (Fig. 5*A*). The number of tdTomato-positive kisspeptin neurons that contacted VNUT-immunopositive fibers and the number of VNUT-immunopositive fiber contact sites per tdTomato-positive cell were significantly higher in the OVX *Kiss1*-tdTomato rats treated with high E2 compared with those of the low E2-treated *Kiss1*-tdTomato rats (*n* = 5 in each group; *p* = 0.045, Fig. 5*B*; *p* = 0.0256, Fig. 5*C*). There was no significant difference in the number of tdTomato-positive kisspeptin neurons between high and low E2-treated groups (Fig. 5*D*). VNUT-immunopositive fibers were rarely found in the POA/MSDB region where GnRH-immunopositive cells were located (Fig. 5*E*), and no contact of VNUT-immunopositive fibers with GnRH-immunopositive cells was found in the POA/MSDB region in OVX + high E2 rats (0 cell/121.7 ± 10.2 GnRH cells, *n* = 3). These results indicate that purinergic neurons project directly to AVPV kisspeptin neurons but not to GnRH neurons.

### Identification of purinergic neurons activated by preovulatory levels of estrogen

**Figure 6.**
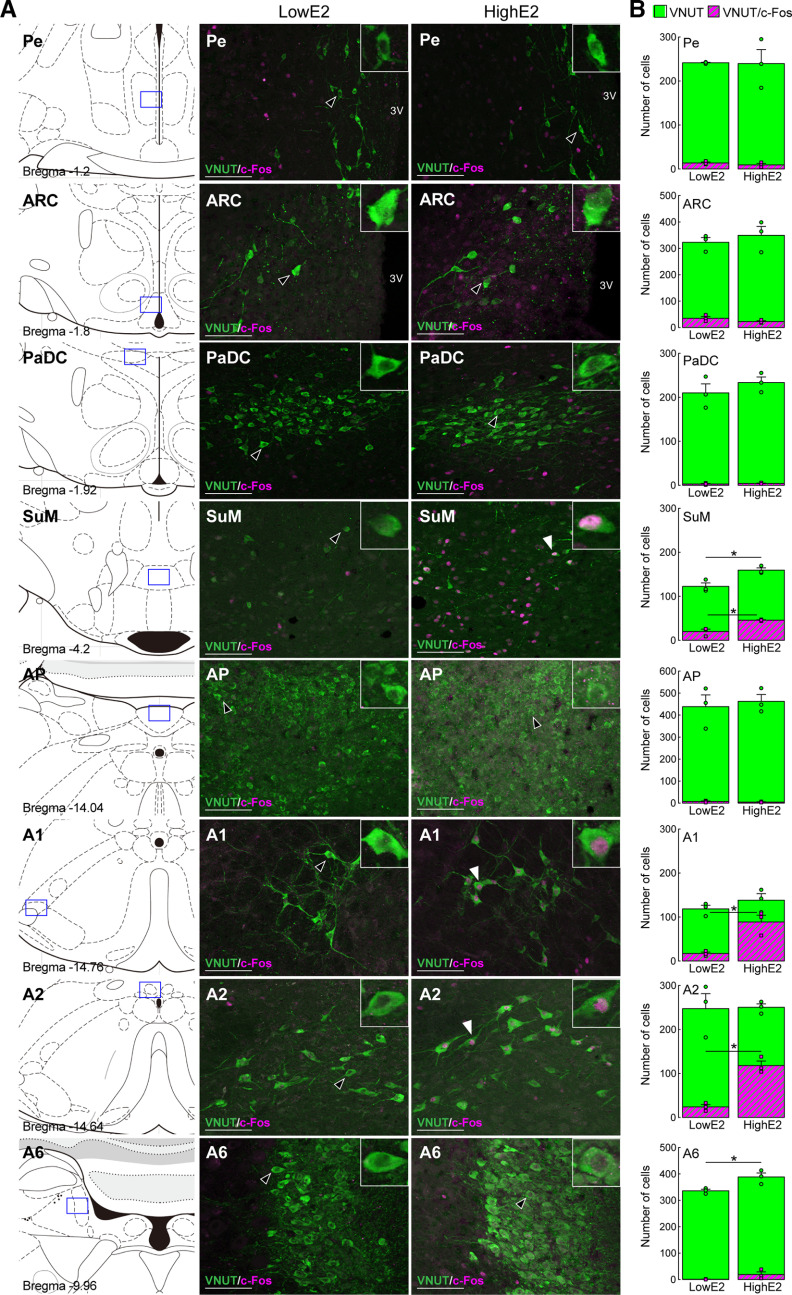
Proestrous levels of E2 activate purinergic neurons in the hindbrain of female rats. ***A***, VNUT-immunoreactive cells were found in the Pe, ARC, PaDC, SuM, AP, and hindbrain noradrenergic nuclei, such as A1, A2, and A6 regions of OVX WT rats treated with low or high E2. ***B***, The number of VNUT-immunopositive cells coexpressing c-Fos was significantly higher in the SuM, A1, and A2 regions in WT OVX + high E2 rats compared with that in OVX + low E2 rats (*n* = 3 in each group): *p* = 0.0095, SuM; *p* = 0.0106, A1; *p* = 0.0012, A2; Student's *t* test. The total number of VNUT-immunopositive cells was significantly higher in the SuM and A6 region of high E2-treated OVX rats compared with that in low E2-treated rats: *p = 0.0164, SuM; *p = 0.0319, A6; Student's *t* test. Data are mean ± SEM. 3 V, Third ventricle. Scale bars, 100 μm.

Figure 6 shows the results of double immunohistochemistry for VNUT and c-Fos (a maker for neural activation) in the hypothalamus and hindbrain of OVX WT rats treated with high E2 or low E2 (*n* = 3 in each group). Many VNUT-immunopositive cell bodies were found in several hypothalamic nuclei, such as the Pe, ARC, PaDC, SuM, and hindbrain noradrenergic nuclei, such as A1, A2, and A6 regions, and AP in both high E2- and low E2-treated OVX rats (Fig. 6*A*). Dense c-Fos immunoreactivity was found in many of the VNUT-immunopositive cells in the SuM, A1, and A2 in OVX + high E2 WT rats, but not in the Pe, ARC, PaDC, AP, and A6. Importantly, the numbers of VNUT-immunopositive cells coexpressing c-Fos was significantly higher in the SuM (45.7 ± 1.0 cells, *p* = 0.0095), A1 (88.7 ± 15.5 cells, *p* = 0.0106), and A2 (118.0 ± 10.3 cells, *p* = 0.0012) of OVX WT rats treated with high E2 compared with those of low E2-treated OVX WT rats (SuM, 19.8 ± 5.4 cells; A1, 17.0 ± 3.2 cells; A2, 24.0 ± 5.2 cells). There was no significant difference in the number of VNUT-immunopositive cells coexpressing c-Fos in the Pe, ARC, PaDC, AP, and A6 between the high E2- and low E2-treated OVX groups (Fig. 6*B*). The total number of VNUT-immunopositive cells was significantly higher in the SuM and A6 of high E2-treated OVX rats compared with that in low E2-treated rats (*p* = 0.0164, SuM; *p* = 0.0319, A6), whereas there was no significant difference in the total number of VNUT-immunopositive cells in the Pe, ARC, PaDC, AP, A1, and A2 between high E2- and low E2-treated groups (Fig. 6*B*).

### Identification of purinergic neurons projecting to the AVPV and coexpression of ERα immunoreactivity in VNUT-immunoreactive purinergic neurons

**Figure 7.**
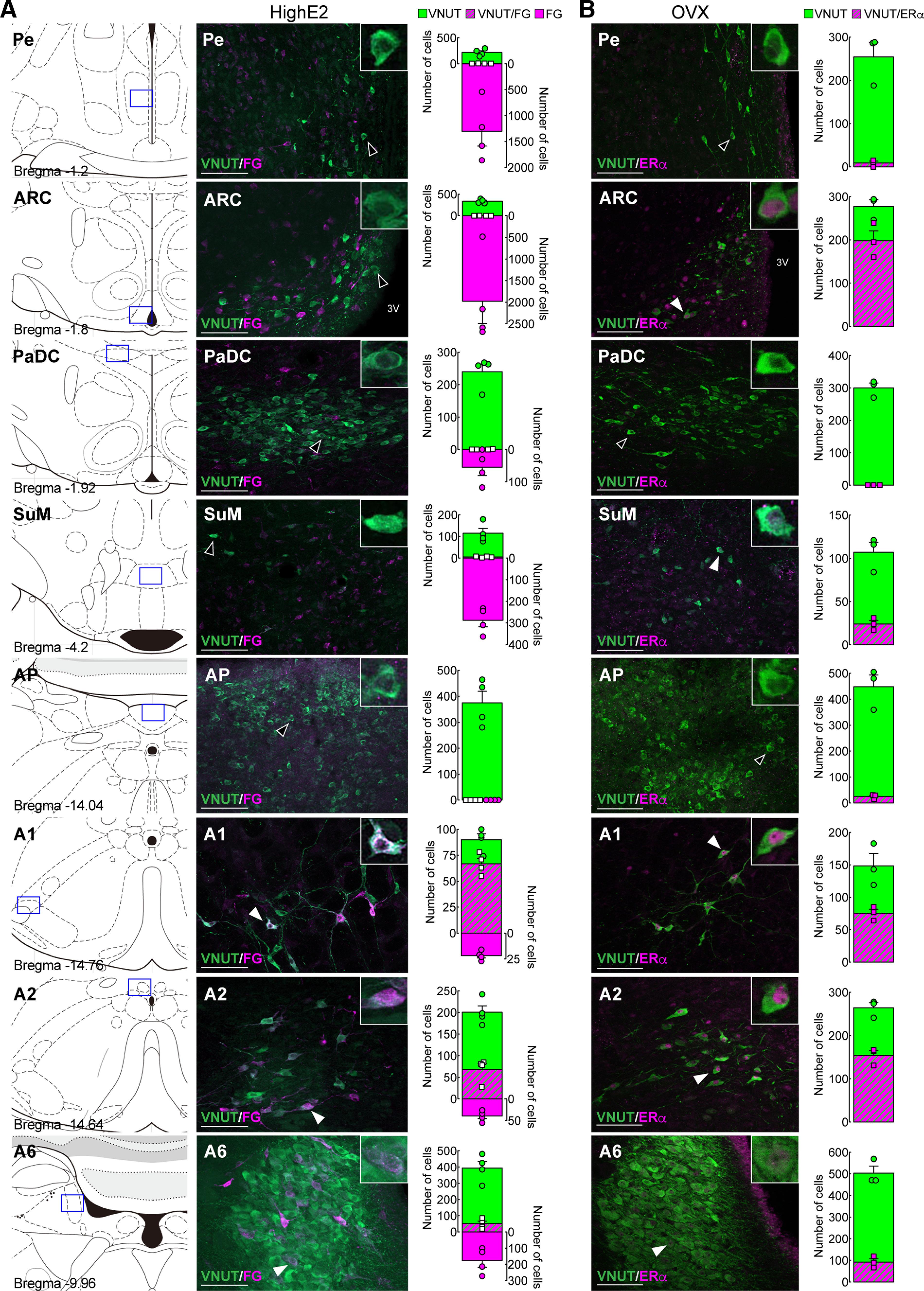
Identification of purinergic neurons projecting to the AVPV and coexpression of ERα immunoreactivity in VNUT-immunoreactive purinergic neurons. ***A***, FG-immunoreactive signals were found in VNUT-positive cells in A1, A2, and A6 in OVX WT rats treated with high E2. No or few FG-immunoreactive signals were found in the Pe, ARC, PaDC, SuM, and AP in VNUT-positive cells of OVX WT rats treated with high E2. ***B***, ERα-immunoreactive signals were found in the majority of VNUT-positive cells in the A1, A2, and ARC and some VNUT-positive cells in the A6 regions and SuM in OVX WT rats. No or few ERα-immunoreactive signals were found in the Pe, PaDC, and AP in VNUT-positive cells of OVX WT rats. Data are mean ± SEM. 3 V, Third ventricle. Scale bars, 100 μm.

Next, we explored purinergic neurons projecting to the AVPV by retrograde tracing and coexpression of ERα in the purinergic neurons. Figure 7*A* shows the results of double immunohistochemistry for VNUT and FG (a retrograde tracer) in the brain, including the hypothalamus and hindbrain, of high E2-treated OVX WT rats injected with FG into the AVPV (*n* = 4). FG-immunoreactive cells were mainly found on the FG-injected side of the ARC and PaDC and on both sides of the SuM, A1, A2, and A6 regions but not in the AP in OVX + high E2 rats (Fig. 7*A*). Quantitative analysis of the FG- and/or VNUT-positive cells in these brain regions ipsilateral to the FG injection site revealed that a majority of VNUT-positive cells showed FG signals in the A1 (66.8 ± 4.3 FG-positive cells/90.0 ± 4.8 VNUT-positive cells, 74.2%), and some VNUT-positive cells showed FG signals in the A2 (68.0 ± 11.0 FG-positive cells/200.5 ± 13.0 VNUT-positive cells, 33.9%) and A6 (50.3 ± 13.2 FG-positive cells/393.5 ± 35.8 VNUT-positive cells, 12.8%). On the other hand, few or no FG signals were found in VNUT-expressing cells in the ARC, PaDC, and SuM. Figure 7*B* shows ERα immunoreactivity in the VNUT-immunoreactive purinergic neurons in the hypothalamus and hindbrain of OVX WT rats (*n* = 3). ERα-immunoreactive signals were found in VNUT-positive cells in the ARC (198.0 ± 18.7 ERα-positive cells/277.0 ± 12.7 VNUT-positive cells, 71.5%), SuM (24.0 ± 3.3 ERα-positive cells/107.0 ± 9.5 VNUT-positive cells, 22.4%), A1 (75.3 ± 5.0 ERα-positive cells/148.3 ± 15.2 VNUT-positive cells, 50.8%), A2 (154.0 ± 9.4 ERα-positive cells/264.3 ± 9.6 VNUT-positive cells, 58.3%), and A6 regions (91.7 ± 12.3 ERα-positive cells/503.3 ± 26.8 VNUT-positive cells, 18.2%) in OVX WT rats (Fig. 7*B*). On the other hand, few or no ERα-immunoreactive signals were found in VNUT-expressing cells in the Pe, PaDC, and AP. These results indicate that A1, A2, A6, ARC, and SuM purinergic neurons could be major sites for E2 action.

### No VNUT-immunoreactive purinergic neurons in the SCN

**Figure 8.**
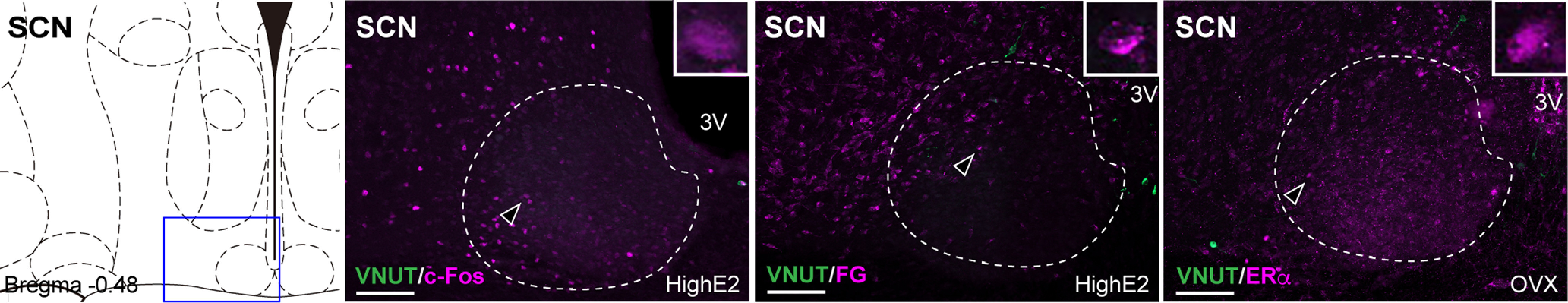
No VNUT-immunoreactive purinergic neurons were observed in the SCN. Double immunohistochemistry for VNUT/c-Fos and VNUT/FG in the SCN of high E2-treated OVX WT rats and for VNUT/ERα in the SCN of OVX WT rats. No VNUT-immunoreactive cells were found in the SCN, but c-Fos- or FG-immunopositive cells were found in the SCN of OVX WT rats treated with high E2. ERα-immunopositive cells were also found in the SCN of OVX WT rats. 3 V, Third ventricle. Scale bars, 100 μm.

We also explored the double immunoreactivities of VNUT and c-Fos, FG, or ERα in the SCN because the timing of the GnRH/LH surge is largely correlated with biological clock signals from the SCN in female rats. Figure 8 shows the results of double immunohistochemistry for VNUT and c-Fos and VNUT and FG in the SCN of OVX WT rats treated with high E2 and for VNUT and ERα in the SCN of OVX WT rats. No VNUT-immunoreactive cell bodies were found in the SCN of OVX WT rats treated with high E2 and OVX WT rats. On the other hand, a large number of c-Fos- or FG-immunoreactive cells without VNUT signals were found in the SCN of high E2-treated OVX WT rats, and a few ERα-immunoreactive cells were found in the SCN of OVX WT rats.

### Brainstem purinergic neurons express DBH, a noradrenergic marker peptide

**Figure 9.**
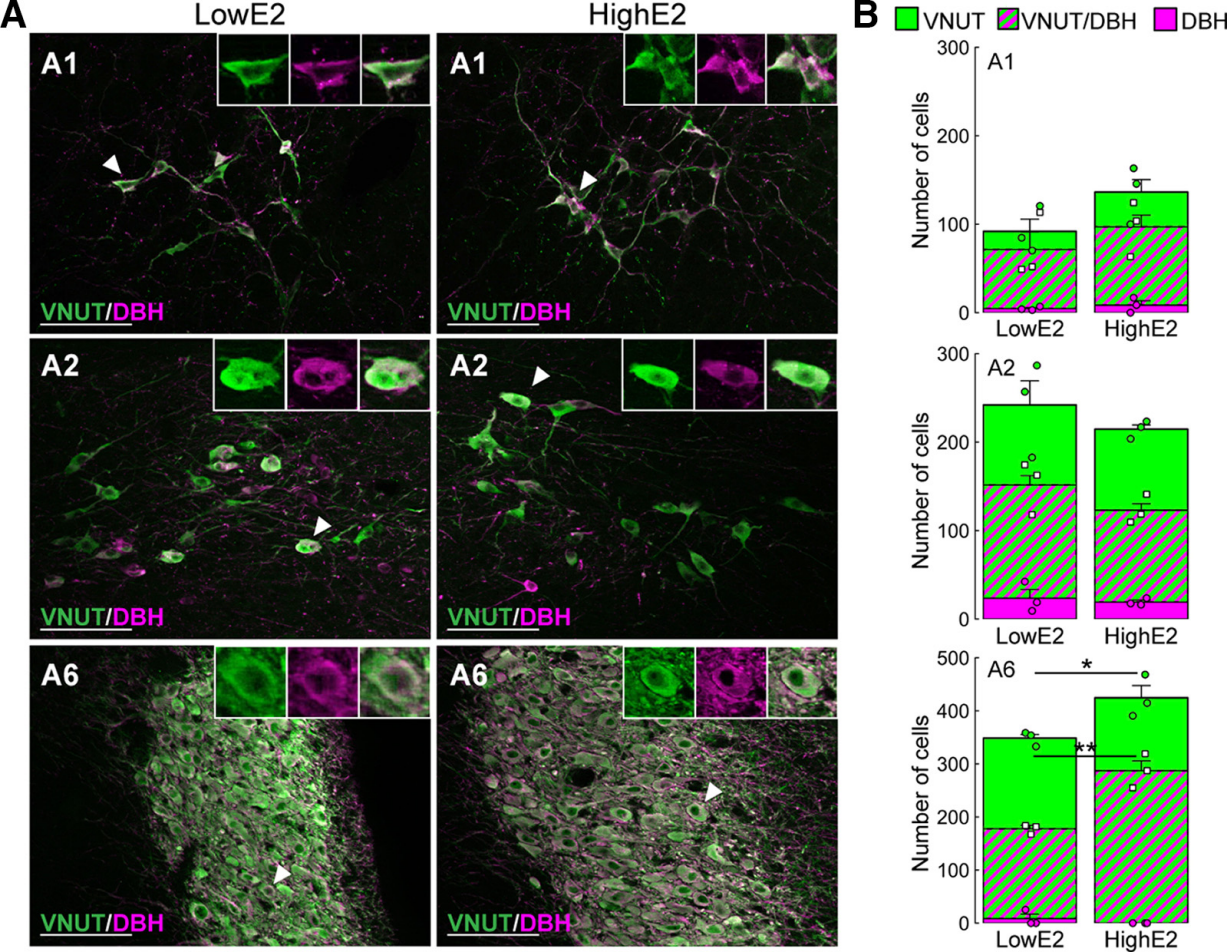
Colocalization of VNUT and DBH (a noradrenergic marker) immunoreactivities in the hindbrains. ***A***, Double immunohistochemistry for VNUT and DBH revealed that most of the DBH-immunopositive cells showed VNUT immunoreactivity in the A1, A2, and A6 regions of WT OVX + high E2 and OVX + low E2 rats. ***B***, The number of VNUT-immunopositive cells and VNUT and DBH double-immunopositive cells in the A6 of OVX + high E2 rats was significantly higher compared with that in OVX + low E2 rats (*n* = 3 in each group): *p = 0.0244, VNUT; **p = 0.0253, VNUT + DBH; Student's *t* test). Data are mean ± SEM. Scale bars, 100 μm.

Figure 9 shows the results of double immunohistochemistry for VNUT and DBH (a marker of noradrenergic neurons) in the hindbrain of OVX WT rats treated with high E2 or low E2. Most of the DBH-immunopositive cells showed VNUT immunoreactivity in the A1, A2, and A6 regions of both high E2- and low E2-treated OVX rats (Fig. 9*A*). Quantitative analysis revealed that the numbers of VNUT-immunopositive cells and VNUT and DBH double-immunopositive cells in the A6 region of OVX + high E2 rats were significantly higher compared with those in OVX + low E2 rats (*n* = 3 in each group; *p* = 0.0253, Fig. 9*B*). There was no significant difference in the numbers of VNUT-immunopositive cells or VNUT and DBH double-immunopositive cells in the A1 and A2 regions between the groups (Fig. 9*B*).

## Discussion

The present study demonstrates that purinergic signaling plays a critical role in GnRH/LH surge generation and consequent ovulation via direct stimulation of AVPV kisspeptin neurons. This is because the administration of a purinergic receptor antagonist into the AVPV region completely blocked estrogen-induced LH surge in OVX rats, and AVPV kisspeptin neurons express P2X2 receptors. Furthermore, administration of ATP into the AVPV region immediately stimulated a surge-like increase in LH release in the morning in WT rats but not in global *Kiss1* KO rats. Our conclusion was further supported by the current *in vitro* study showing that ATP immediately activated AVPV kisspeptin neuron-derived cells (mHypoA-51) via P2X receptors expressed in the cells. Importantly, the number of varicosity-like P2X2 receptor-immunoreactive signals surrounding AVPV kisspeptin neurons was significantly higher in OVX rats primed with the proestrous level of estrogen than in OVX rats with the diestrous level of estrogen, indicating that an increase in the circulating level of estrogen along with follicular development enhances the responsiveness of AVPV kisspeptin neurons to the purinergic ligand ATP. These results indicate that ATP-P2X2 receptor signaling is a possible mediator of estrogen-induced activation of AVPV kisspeptin neurons in their generation of the GnRH/LH surge and consequent triggering of ovulation. Indeed, the current P2RX antagonism in the AVPV largely attenuated spontaneous proestrous LH surge and consequently reduced the ovulation rate in ovary-intact rats. To our best knowledge, this is the first finding that central ATP-purinergic signaling plays a critical role in induction of GnRH/LH surge and consequent ovulation via stimulation of AVPV kisspeptin neurons. This finding offers a new avenue to develop therapeutics for reproductive disorders, such as ovulation disorders in humans as well as in livestock.

Purinergic/noradrenergic neurons originating in the A1 and A2 regions of the lower brainstem are the most likely source of ATP involved in GnRH/LH surge induction. This is because the proestrous level of estrogen markedly increased the number of VNUT (a marker of purinergic neurons)-positive cells expressing c-Fos (a marker of neural activation) in these nuclei in OVX female rats just before the onset of LH surge, and most of the DBH-positive cells in these nuclei expressed VNUT. Notably, the current retrograde tracing study suggests that a majority of A1 and some A2 VNUT-positive purinergic neurons directly project to the AVPV region of female rats. Furthermore, the proestrous level of estrogen significantly increased the number of AVPV kisspeptin neurons surrounded by VNUT-positive varicosity-like structures and the number of structures per AVPV kisspeptin neuron. These findings indicate that an increase in circulating estrogen from mature follicles activates brainstem purinergic/noradrenergic neurons projecting to AVPV kisspeptin neurons and increases the responsiveness of the kisspeptin neurons to the purinergic ligand. Importantly, most of the purinergic/noradrenergic neurons in the A1 and A2 regions express ERα, indicating that circulating estrogen may directly activate brainstem purinergic/noradrenergic neurons to trigger the GnRH/LH surge. These findings suggest that ATP from brainstem purinergic neurons is a key signal in triggering the GnRH/LH surge and consequent ovulation, as shown by the blockade of LH surge and ovulation by P2RX antagonism in the AVPV region. Noradrenaline might be coreleased with ATP but may not be essential for the GnRH/LH surge, because α1-adrenergic receptor, a major central stimulatory noradrenergic receptor, was not found on AVPV kisspeptin neurons in estrogen-primed OVX rats (Uchôa et al., 2016). On the other hand, noradrenaline may facilitate the action of ATP in an autocrine manner to enhance P2X2 receptor-mediated kisspeptin release from AVPV neurons because noradrenaline administration to the POA enlarged the LH surge (Szawka et al., 2013), whereas administration of an α1-adrenergic receptor antagonist to the POA attenuated the LH surge in female rats (Weesner et al., 1993). Furthermore, brainstem noradrenergic neurons expressed c-Fos before an estrogen-induced LH surge in female rats (Szawka et al., 2013); and noradrenaline release increased in both the MPOA (Mohankumar et al., 1994) and the medial basal hypothalamus (ThyagaRajan et al., 1995) coinciding with the afternoon LH surge in proestrous rats. ATP is reportedly often coreleased with noradrenaline from the nerve terminals of catecholaminergic neurons of brain slices that include region A6 (Poelchen et al., 2001). Together, these findings suggest that ATP coreleased with noradrenaline is a possible neurotransmitter triggering the GnRH/LH surge via direct activation of AVPV kisspeptin neurons through purinergic receptors expressed in the neurons. This notion is supported by the current findings that AVPV P2RX antagonism completely blocked estrogen-induced LH surge and largely attenuated ovulation. In addition, non-noradrenergic purinergic neurons in the A1 or A2 regions may also participate in GnRH/LH surge induction because many VNUT-positive/DBH-negative cells were located in these nuclei.

Purinergic neurons in the SuM could be indirectly involved in inducing the GnRH/LH surge because the number of VNUT-positive cells and cells coexpressing c-Fos and VNUT in the SuM significantly increased just before the LH surge, but few FG signals were found in the SuM VNUT-positive cells in female rats. SuM neurons reportedly project to the hippocampus and MSDB region (Borhegyi et al., 1998; Chen et al., 2020). Meanwhile, the current study shows that VNUT-positive fibers were rare in the MSDB in the vicinity of GnRH cell bodies and that ERα was not found in SuM VNUT-positive cells. Further studies are required to clarify whether SuM purinergic neurons and nonadrenergic purinergic neurons are involved in GnRH/LH surge induction.

Interestingly, the current AVPV ATP administration slightly but not significantly increased LH release in OVX + high E2 *Kiss1* KO rats. This suggests that non-kisspeptin neurons in the AVPV could be an ATP action site to stimulate GnRH/LH release. Previous studies reported that purinergic receptors, such as P2X2, P2X4, P2X5, P2X6, and P2X7, are expressed in mouse GnRH neurons (Jiqiang et al., 2009; Vastagh et al., 2016), and P2X2 and P2X4 receptors were expressed in GnRH neurons of rhesus monkeys (Terasawa et al., 2005). Thus, it is possible that the current AVPV ATP administration may have directly activated GnRH neurons to induce a slight increase in GnRH/LH secretion in *Kiss1* KO rats.

In rodents, SCN arginine vasopressin (AVP) neurons have been suggested to be an important component of the biological clock that controls the timing of LH surge that occurs in the afternoon (Funabashi et al., 1999). Indeed, administration of an anti-AVP antibody into the POA attenuated the endogenous LH surge in proestrous rats (Funabashi et al., 1999), and AVPV kisspeptin neurons express V1A, a vasopressin receptor. Furthermore, SCN AVP neurons project to AVPV kisspeptin neurons in hamsters (Williams et al., 2011). Consistent with the latter, the current study showed VNUT-negative neural projections from the SCN to the AVPV region, and c-Fos-positive cells without VNUT signals were evident in the SCN of high E2-treated OVX rats. Moreover, ERα-positive VNUT-negative cells were found in the SCN of OVX rats. Notably, in the present study, no VNUT-immunopositive cells were found in the SCN, indicating that SCN AVP neurons are not the source of purinergic ATP that triggers the GnRH/LH surge. Thus, it is likely that SCN AVP neurons and purinergic neurons are independently involved in the E2-induced GnRH/LH surge.

Notably, diestrous levels of E2 increased AVPV *Kiss1* expression to levels similar to those shown in OVX rats treated with proestrous levels of E2 (Kinoshita et al., 2005; Adachi et al., 2007), whereas proestrous levels of E2 but not diestrous levels of E2 activate AVPV kisspeptin neurons before the LH surge in OVX rats (Adachi et al., 2007). The present study showed that high E2 treatment significantly activated A1 and A2 purinergic neurons just before the occurrence of the LH surge, and ERα expression was evident in these purinergic neurons in OVX rats. These findings suggest that the proestrous levels of E2 may directly activate A1 and A2 purinergic neurons to trigger the GnRH/LH surge via activation of AVPV kisspeptin neurons in female rats.

A recent study showed that P2rx5, a P2X receptor, was evident in mouse AVPV kisspeptin neurons and that estrogen upregulated *P2rx5* expression (Stephens and Kauffman, 2021), suggesting that ATP-P2RX5 signaling in AVPV kisspeptin neurons may be involved in GnRH/LH surge generation in mice. However, the current transcriptome analysis of rat AVPV kisspeptin neurons showed an estrogen-dependent increase in P2rx2 gene expression but failed to detect the expression of other purinergic receptor genes regardless of the estrogen milieu. Thus, there may be a species difference in the type of purinergic receptors in AVPV kisspeptin neurons.

In conclusion, our current findings suggest that ATP-purinergic signaling in AVPV kisspeptin neurons plays a critical role in induction of the GnRH/LH surge and consequent ovulation in female rats. Specifically, ATP may directly stimulate AVPV kisspeptin neurons to trigger GnRH/LH surge in the presence of preovulatory levels of estrogen. We also suggest that the preovulatory level of estrogen activates A1 and A2 purinergic/noradrenaline neurons, which would be a major source of ATP-purinergic signals. The present study provides the first report demonstrating that brainstem ATP-purinergic signaling triggers ovulation via direct activation of forebrain kisspeptin neurons in mammals. These findings may contribute to new treatments for ovulation disorders in women as well as livestock.

## References

[B1] Adachi S, Yamada S, Takatsu Y, Matsui H, Kinoshita M, Takase K, Sugiura H, Ohtaki T, Matsumoto H, Uenoyama Y, Tsukamura H, Inoue K, Maeda KI (2007) Involvement of anteroventral periventricular metastin/kisspeptin neurons in estrogen positive feedback action on luteinizing hormone release in female rats. J Reprod Dev 53:367–378. 10.1262/jrd.18146 17213691

[B2] Assadullah I, Ieda N, Kawai N, Ishii H, Ihara K, Inoue N, Uenoyama Y, Tsukamura H (2018) Coexpression of the calcitonin receptor gene in the hypothalamic kisspeptin neurons in female rats. Reprod Med Biol 17:164–172. 10.1002/rmb2.12085 29692674 PMC5902471

[B3] Belsham DD, Cai F, Cui H, Smukler SR, Salapatek AM, Shkreta L (2004) Generation of a phenotypic array of hypothalamic neuronal cell models to study complex neuroendocrine disorders. Endocrinology 145:393–400. 10.1210/en.2003-0946 14551229

[B4] Borhegyi Z, Maglóczky Z, Acsády L, Freund TF (1998) The supramammillary nucleus innervates cholinergic and gabaergic neurons in the medial septum-diagonal band of Broca complex. Neuroscience 82:1053–1065. 10.1016/s0306-4522(97)00301-1 9466429

[B5] Cagampang FR, Maeda KI, Yokoyama A, Ota K (1990) Effect of food deprivation on the pulsatile LH release in the cycling and ovariectomized female rat. Horm Metab Res 22:269–272. 10.1055/s-2007-1004900 2347540

[B6] Chen S, He L, Huang AJ, Boehringer R, Robert V, Wintzer ME, Polygalov D, Weitemier AZ, Tao Y, Gu M, Middleton SJ, Namiki K, Hama H, Therreau L, Chevaleyre V, Hioki H, Miyawaki A, Piskorowski RA, McHugh TJ (2020) A hypothalamic novelty signal modulates hippocampal memory. Nature 586:270–274. 10.1038/s41586-020-2771-1 32999460

[B7] Clarkson J, Han SY, Piet R, McLennan T, Kane GM, Ng J, Porteous RW, Kim JS, Colledge WH, Iremonger KJ, Herbison AE (2017) Definition of the hypothalamic GnRH pulse generator in mice. Proc Natl Acad Sci USA 114:E10216–E10223. 10.1073/pnas.1713897114 29109258 PMC5703322

[B8] De Roux N, Genin E, Carel JC, Matsuda F, Chaussain JL, Milgrom E (2003) Hypogonadotropic hypogonadism due to loss of function of the KiSS1-derived peptide receptor GPR54. Proc Natl Acad Sci USA 100:10972–10976. 10.1073/pnas.1834399100 12944565 PMC196911

[B9] Franceschini I, Lomet D, Cateau M, Delsol G, Tillet Y, Caraty A (2006) Kisspeptin immunoreactive cells of the ovine preoptic area and arcuate nucleus coexpress estrogen receptor alpha. Neurosci Lett 401:225–230. 10.1016/j.neulet.2006.03.039 16621281

[B10] Funabashi T, Aiba S, Sano A, Shinohara K, Kimura F (1999) Intracerebroventricular injection of arginine-vasopressin V1 receptor antagonist attenuates the surge of luteinizing hormone and prolactin secretion in proestrous rats. Neurosci Lett 260:37–40. 10.1016/s0304-3940(98)00940-9 10027694

[B11] Jiqiang F, Qiang Y, Wei G, Cheng H, Geoffrey B, Zhenghua X (2009) P2X receptors are expressed on neurons containing luteinizing hormone-releasing hormone in the mouse hypothalamus. Neurosci Lett 458:32–36. 10.1016/j.neulet.2009.04.017 19442872

[B12] Khakh BS, North RA (2012) Neuromodulation by extracellular ATP and P2X receptors in the CNS. Neuron 76:51–69. 10.1016/j.neuron.2012.09.024 23040806 PMC4064466

[B13] Kinoshita M, Tsukamura H, Adachi S, Matsui H, Uenoyama Y, Iwata K, Yamada S, Inoue K, Ohtaki T, Matsumoto H, Maeda KI (2005) Involvement of central metastin in the regulation of preovulatory luteinizing hormone surge and estrous cyclicity in female rats. Endocrinology 146:4431–4436. 10.1210/en.2005-0195 15976058

[B14] Lapatto R, Pallais JC, Zhang D, Chan YM, Mahan A, Cerrato F, Le WW, Hoffman GE, Seminara SB (2007) Kiss1^-/-^ mice exhibit more variable hypogonadism than Gpr54^-/-^ mice. Endocrinology 148:4927–4936. 10.1210/en.2007-0078 17595229

[B15] Matsuda F, Nakatsukasa K, Suetomi Y, Naniwa Y, Ito D, Inoue N, Wakabayashi Y, Okamura H, Maeda KI, Uenoyama Y, Tsukamura H, Ohkura S (2015) The luteinising hormone surge-generating system is functional in male goats as in females: involvement of kisspeptin neurones in the medial preoptic area. J Neuroendocrinol 27:57–65. 10.1111/jne.12235 25367275

[B16] Messager S, Chatzidaki EE, Ma D, Hendrick AG, Zahn D, Dixon J, Thresher RR, Malinge I, Lomet D, Carlton MB, Colledge WH, Caraty A, Aparicio SA (2005) Kisspeptin directly stimulates gonadotropin-releasing hormone release via G protein-coupled receptor 54. Proc Natl Acad Sci USA 102:1761–1766. 10.1073/pnas.0409330102 15665093 PMC545088

[B17] Minabe S, Deura C, Ikegami K, Goto T, Sanbo M, Hirabayashi M, Inoue N, Uenoyama Y, Maeda KI, Tsukamura H (2015) Pharmacological and morphological evidence of AMPK-mediated energy sensing in the lower brain stem ependymocytes to control reproduction in female rodents. Endocrinology 156:2278–2287. 10.1210/en.2014-2018 25822714 PMC4430616

[B18] Mittelman-Smith MA, Wong AM, Kathiresan AS, Micevych PE (2015) Classical and membrane-initiated estrogen signaling in an in vitro model of anterior hypothalamic kisspeptin neurons. Endocrinology 156:2162–2173. 10.1210/en.2014-1803 25730107 PMC4430613

[B19] Mohankumar PS, Thyagarajan S, Quadri SK (1994) Correlations of catecholamine release in the medial preoptic area with proestrous surges of luteinizing hormone and prolactin: effects of aging. Endocrinology 135:119–126. 10.1210/endo.135.1.8013343 8013343

[B20] Nagae M, Uenoyama Y, Okamoto S, Tsuchida H, Ikegami K, Goto T, Majarune S, Nakamura S, Sanbo M, Hirabayashi M, Kobayashi K, Inoue N, Tsukamura H (2021) Direct evidence that KNDy neurons maintain gonadotropin pulses and folliculogenesis as the GnRH pulse generator. Proc Natl Acad Sci USA 118:e2009156118. 33500349 10.1073/pnas.2009156118 PMC7865162

[B21] Park MK, Wakabayashi K (1986) Preparation of a monoclonal antibody to common amino acid sequence of LHRH and its application. Endocrinol Jpn 33:257–272. 10.1507/endocrj1954.33.257 3530729

[B22] Paxinos G, Watson C (2008) The rat brain in stereotaxic coordinates, Ed 6. San Diego: Academic. 10.1016/0165-0270(80)90021-7 6110810

[B23] Poelchen W, Sieler D, Wirkner K, Illes P (2001) Co-transmitter function of ATP in central catecholaminergic neurons of the rat. Neuroscience 102:593–602. 10.1016/s0306-4522(00)00529-7 11226696

[B24] Seminara SB, Messager S, Chatzidaki EE, Thresher RR, Acierno JS, Shagoury JK, Bo-Abbas Y, Kuohung W, Schwinof KM, Hendrick AG, Zahn D, Dixon J, Kaiser UB, Slaugenhaupt SA, Gusella JF, O'Rahilly S, Carlton MB, Crowley WF, Aparicio SA, Colledge WH (2003) The GPR54 gene as a regulator of puberty. N Engl J Med 349:1614–1627. 10.1056/NEJMoa035322 14573733

[B25] Smith JT, Cunningham MJ, Rissman EF, Clifton DK, Steiner RA (2005) Regulation of Kiss1 gene expression in the brain of the female mouse. Endocrinology 146:3686–3692. 10.1210/en.2005-0488 15919741

[B26] Smith JT, Li Q, Pereira A, Clarke IJ (2009) Kisspeptin neurons in the ovine arcuate nucleus and preoptic area are involved in the preovulatory luteinizing hormone surge. Endocrinology 150:5530–5538. 10.1210/en.2009-0712 19819940

[B27] Smith JT, Shahab M, Pereira A, Pau KY, Clarke IJ (2010) Hypothalamic expression of KISS1 and gonadotropin inhibitory hormone genes during the menstrual cycle of a non-human primate1. Biol Reprod 83:568–577. 10.1095/biolreprod.110.085407 20574054 PMC2957156

[B28] Stephens SB, Kauffman AS (2021) Estrogen regulation of the molecular phenotype and active translatome of AVPV kisspeptin neurons. Endocrinology 162:1–20. 10.1210/endocr/bqab080 PMC8286094 33856454

[B29] Szawka RE, Poletini MO, Leite CM, Bernuci MP, Kalil B, Mendonça LB, Carolino RO, Helena CV, Bertram R, Franci CR, Anselmo-Franci JA (2013) Release of norepinephrine in the preoptic area activates anteroventral periventricular nucleus neurons and stimulates the surge of luteinizing hormone. Endocrinology 154:363–374. 10.1210/en.2012-1302 23150494 PMC3529374

[B30] Terasawa E, Keen KL, Grendell RL, Golos TG (2005) Possible role of 5′-adenosine triphosphate in synchronization of Ca^2+^ oscillations in primate luteinizing hormone-releasing hormone neurons. Mol Endocrinol 19:2736–2747. 10.1210/me.2005-0034 15994201

[B31] ThyagaRajan S, MohanKumar PS, Quadri SK (1995) Cyclic changes in the release of norepinephrine and dopamine in the medial basal hypothalamus: effects of aging. Brain Res 689:122–128. 10.1016/0006-8993(95)00551-z 8528695

[B32] Tsukamura H (2022) Kobayashi Award 2019: the neuroendocrine regulation of the mammalian reproduction. Gen Comp Endocrinol 315:113755. 10.1016/j.ygcen.2021.113755 33711315

[B33] Uchôa ET, Rodrigues JA, Plant TM, Elias LL, Ribeiro AB, Anselmo-Franci JA, Leite CM, Kalil B, Poletini MO, Cardoso TS, Carolino RO (2016) The increase in signaling by kisspeptin neurons in the preoptic area and associated changes in clock gene expression that trigger the LH surge in female rats are dependent on the facilitatory action of a noradrenaline input. Endocrinology 157:323–335. 10.1210/en.2015-1323 26556532

[B34] Uenoyama Y, Nakamura S, Hayakawa Y, Ikegami K, Watanabe Y, Deura C, Minabe S, Tomikawa J, Goto T, Ieda N, Inoue N, Sanbo M, Tamura C, Hirabayashi M, Maeda KI, Tsukamura H (2015) Lack of pulse and surge modes and glutamatergic stimulation of luteinising hormone release in Kiss1 knockout rats. J Neuroendocrinol 27:187–197. 10.1111/jne.12257 25582792

[B35] Uenoyama Y, Inoue N, Nakamura S, Tsukamura H (2021) Kisspeptin neurons and estrogen–estrogen receptor α signaling: unraveling the mystery of steroid feedback system regulating mammalian reproduction. Int J Mol Sci 22:9229. 10.3390/ijms22179229 34502135 PMC8430864

[B36] Vastagh C, Rodolosse A, Solymosi N, Liposits Z (2016) Altered expression of genes encoding neurotransmitter receptors in GnRH neurons of proestrous mice. Front Cell Neurosci 10:230. 27774052 10.3389/fncel.2016.00230 PMC5054603

[B37] Watanabe Y, Uenoyama Y, Suzuki J, Takase K, Suetomi Y, Ohkura S, Inoue N, Maeda KI, Tsukamura H (2014) Oestrogen-induced activation of preoptic kisspeptin neurones may be involved in the luteinising hormone surge in male and female Japanese monkeys. J Neuroendocrinol 26:909–917. 10.1111/jne.12227 25283748

[B38] Watanabe Y, Ikegami K, Ishigaki R, Ieda N, Uenoyama Y, Maeda KI, Tsukamura H, Inoue N (2017) Enhancement of the luteinising hormone surge by male olfactory signals is associated with anteroventral periventricular Kiss1 cell activation in female rats. J Neuroendocrinol 29:e12505. 10.1111/jne.12505 28699305

[B39] Weesner GD, Krey LC, Pfaff DW (1993) α1 adrenergic regulation of estrogen-induced increases in luteinizing hormone-releasing hormone mRNA levels and release. Mol Brain Res 17:77–82. 10.1016/0169-328x(93)90075-z 8381912

[B40] Williams WP, Jarjisian SG, Mikkelsen JD, Kriegsfeld LJ (2011) Circadian control of kisspeptin and a gated GNRH response mediate the preovulatory luteinizing hormone surge. Endocrinology 152:595–606. 10.1210/en.2010-0943 21190958 PMC3037169

